# Utilizing redox-sensitive GFP fusions to detect *in vivo* redox changes in a genetically engineered prokaryote

**DOI:** 10.1016/j.redox.2019.101280

**Published:** 2019-07-20

**Authors:** Wilhad Hans Reuter, Thorsten Masuch, Na Ke, Marine Lenon, Meytal Radzinski, Vu Van Loi, Guoping Ren, Paul Riggs, Haike Antelmann, Dana Reichmann, Lars I. Leichert, Mehmet Berkmen

**Affiliations:** a New England Biolabs, 240 County Road, Ipswich, MA, 01938, USA; b Ruhr University Bochum, Institute of Biochemistry and Pathobiochemistry, Microbial Biochemistry, Universitätsstr. 150, 44780, Bochum, Germany; c The Hebrew University of Jerusalem, The Alexander Silberman Institute of Life Sciences, Safra Campus Givat Ram, Jerusalem, 91904, Israel; d Institute for Biology-Microbiology, Freie Universität Berlin, 14195, Berlin, Germany

**Keywords:** roGFP, Redox, SHuffle, Disulfide bond formation

## Abstract

Understanding the *in vivo* redox biology of cells is a complex albeit important biological problem. Studying redox processes within living cells without physical disruption or chemical modifications is essential in determining the native redox states of cells. In this study, the previously characterized reduction-oxidation sensitive green fluorescent protein (roGFP2) was used to elucidate the redox changes of the genetically engineered *Escherichia coli* strain, SHuffle. SHuffle cells were demonstrated to be under constitutive oxidative stress and responding transcriptionally in an OxyR-dependent manner. Using roGFP2 fused to either glutathione (GSH)- or hydrogen peroxide (H_2_O_2_)- sensitive proteins (glutaredoxin 1 or Orp1), the cytosolic redox state of both wild type and SHuffle cells based on GSH/GSSG and H_2_O_2_ pools was measured. These probes open the path to *in vivo* studies of redox changes and genetic selections in prokaryotic hosts.

## 1 Introduction

*Escherichia coli* is one of the best characterized prokaryotic host for protein expression and is used for biotechnological research worldwide [1]. Understanding the biological processes that govern protein production in *E. coli* will improve both expression levels and stability of recombinant proteins.

Reductases in the cytosol of wild type *E. coli* maintain cysteine in its reduced state, which makes the cytosol of common *E. coli* expression strains not amicable for the production of disulfide-bonded proteins. Disulfide bonds are formed by the oxidation of thiol groups; in *E. coli,* their formation takes place almost exclusively in the periplasm and is catalyzed by a devoted machinery [2]. However, this system is not present in the cytosol and additional genetic engineering is necessary to facilitate the formation of disulfide bonds on recombinant proteins expressed in this compartment.

In order to generate an oxidative cytosolic compartment for the expression of disulfide bonded proteins, an *E. coli* strain named SHuffle was genetically engineered. SHuffle strains have their disulfide bond reductase pathways disrupted, permitting the oxidation of thiols within cysteines residues, resulting in disulfide bonds. Further improvements to the fidelity of disulfide bond formation were achieved by the genomic expression of the disulfide bond isomerase DsbC, in the cytoplasm [3]. Genetic removal of thioredoxin reductase (*trxB*) and glutathione reductase (*gor*) results in cell lethality, which is suppressed by the mutation in the typical 2-Cys alkyl hydroperoxide reductase, *ahpC* [4]. The resulting mutant protein AhpC* has lost its ability to reduce hydrogen peroxide (H_2_O_2_) and has instead gained the function to reduce glutathionylated Glutaredoxin 1 (*grxA*) [5]. Grx1 as reduced by AhpC*, can contribute sufficient reducing power to re-cycle certain essential proteins such as ribonucleotide reductase (*rnr*) back to their reduced states to enable growth [6], while the thioredoxin pathway remains in its oxidized state. It has been suggested that the oxidized Trx1 and Trx2 can oxidize cytoplasmic expressed alkaline phosphatase, 80% and 20% respectively [7], though such studies have yet to be conducted in SHuffle cells.

The native disulfide bond forming pathway of wild type *E. coli* cells has been thoroughly studied [8,9], yet our understanding of the ‘oxidative cytoplasm’ in the SHuffle Δ*trxB*, Δ*gor*, ahpC* + cytoplasmic DsbC genetic background remains poor, especially *in vivo*. In order to expand our understanding of the *in vivo* state of such genetically modified strains, a genetically-encoded probe that can report the redox state of the cytoplasm is useful.

**Fig. 1:**
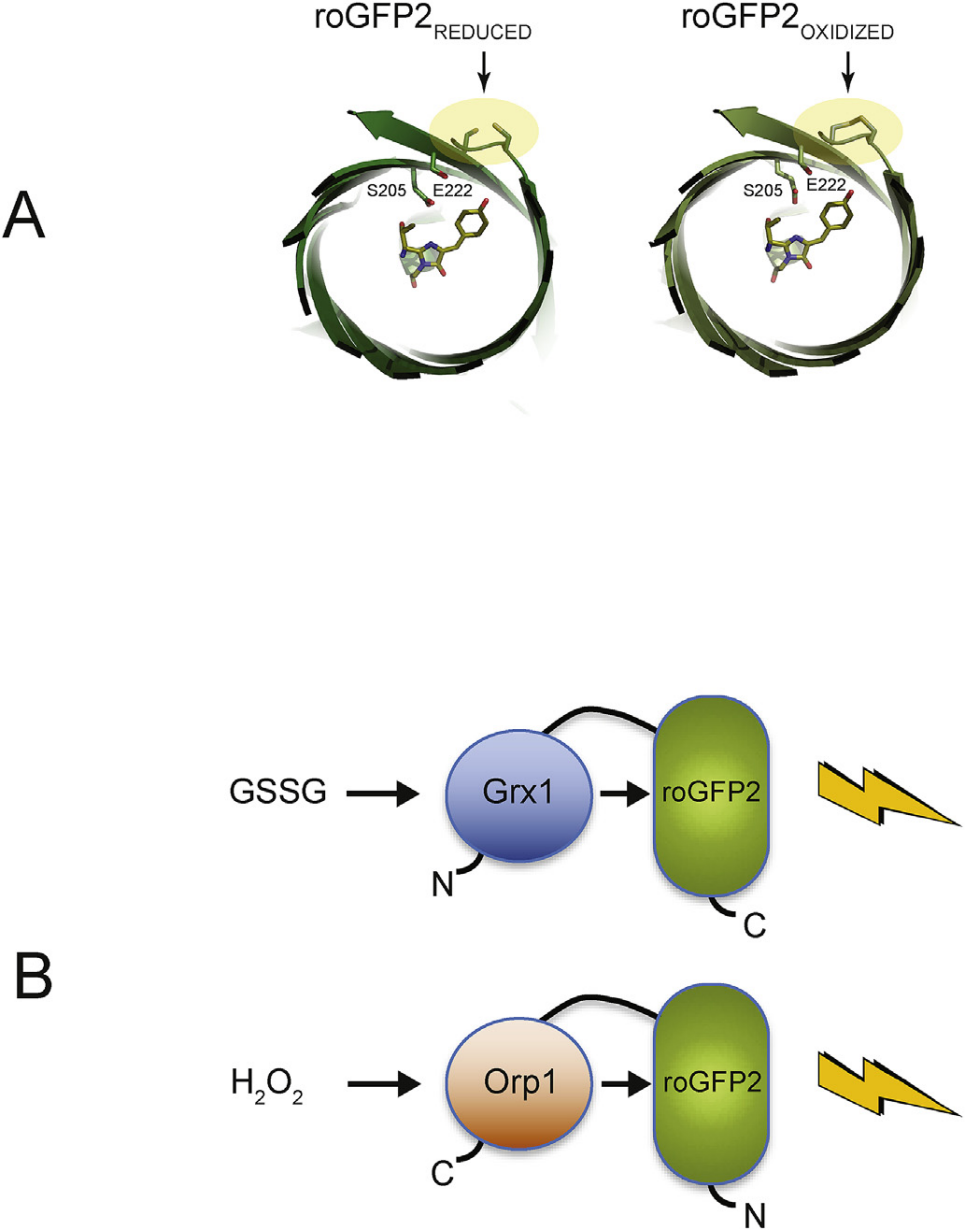
**Molecular mechanism of roGFP2 fusions.** A) Models of reduced (PDB 1JC0) and oxidized (PDB 1JC1) roGFP2. The engineered cysteines S147C and Q204C are highlighted in yellow. The formation of the disulfide bond affects the interaction of the residues S206 and E222 with the chromophore. B) Schematic representation of Grx1-roGFP2 and roGFP2-Orp1 fusion probes. Arrows indicate the redox interactions between the redox active compounds and the probes, resulting in fluorescence. The amino (N) and carboxyl (C) termini are indicated. (For interpretation of the references to colour in this figure legend, the reader is referred to the Web version of this article.)

Within the last decade, significant progress has been made in our understanding of the *in vivo* intracellular redox changes, with the use of redox-sensitive green fluorescent protein (GFP) variants, such as rxYFP [10] and roGFP [11]. roGFP2 is an engineered version of enhanced GFP with two mutations (S147C, Q204C) that can result in a disulfide bond between the engineered cysteines, under favorable oxidative conditions [12]. The formation of a disulfide bond between the engineered cysteines induces a conformational shift that pulls tyrosine 66 away from the chromophore, shifting the chromophore excitation spectra (Fig. 1A) [12]. The ratio in emission at 510 nm when excited at both 405 and 488 nm can then be used to infer the redox state of the environment in which roGFP2 is expressed.

The majority of studies using redox-sensitive fluorescent GFPs have focused on the study of the redox state of eukaryotic cells [[13], [14]], while only a few studies have been conducted in prokaryotic cells, for example *Salmonella* [15], *Corynebacterium* [16], pathogenic bacteria such as *Mycobacterium*, *Listeria* [17] and *Staphylococcus* [18], characterization of the NAD+/NADH pools in *Escherichia coli* [19] and in phagocytosed *E. coli* [20]. In this study, the fluorescent reporter roGFP2, fused to two different redox-sensitive adapters, was used to understand the redox state and changes in SHuffle cells. The fusion of human glutaredoxin-1 to roGFP2 (Grx1-roGFP2) increases the speed of roGFP2 oxidation by glutathione disulfide (GSSG) by 100,000-fold [21]. Similarly, fusing the yeast glutathione peroxidase Orp1 to roGFP2 resulted in a H_2_O_2_ specific redox biosensor (Fig. 1B) [22]. A detailed *in vitro* study of roGFP2 constructs revealed that roGFP2-Orp1 and Grx1-roGFP2 exhibit high specificity towards H_2_O_2_ and oxidized glutathione, respectively. Specifically, roGFP2-Orp1 was highly selective at concentrations of up to 100 μM H_2_O_2_ when compared to the other probes [23,24].

In this study, the functionality of roGFP2-fusion constructs to report the redox state of the cytoplasm of a genetically engineered *E. coli* strain, SHuffle, was confirmed. Using fluorescence-based studies, it was demonstrated that SHuffle cells are under constant H_2_O_2_-based oxidative stress and accumulate elevated levels of GSSG during the growth of the cells. These probes provide valuable insight into the redox changes of an *E. coli* expression strain and thus our knowledge marks a starting point for the engineering of novel, more effective protein expression strains.

## 2 Materials and methods

*E. coli* strains and plasmids: Bacterial strains and plasmids used in this work are described in Table 1 and were constructed using standard molecular and genetic techniques [25].

**Table 1:** Bacterial strains and plasmids utilized in this study.

Strains	Relevant Genotype	Source
NEB express T7	*E. coli BL21 fhuA2 lacZ*::*T7 gene1 [lon] ompT gal sulA11 R(mcr-73*::*miniTn10--*Tet^S^*)2 [dcm] R(zgb-210::Tn10--*Tet^S^*) endA1* Δ*(mcrC-mrr)114::IS10*	NEB cat# C2566
SHuffle express T7	*E. coli BL21 fhuA2 lacZ::T7 gene1 [lon] ompT ahpC gal λatt::pNEB3-r1-cDsbC* (Spec^R^, *lacI*^*q*^) *ΔtrxB sulA11 R(mcr-73::miniTn10--*Tet^S^*)2 [dcm] R(zgb-210::Tn10 --*Tet^S^*) endA1 Δgor Δ(mcrC-mrr)114::IS10*	NEB cat# C3029
MB2938	C2566 + pZE1psoxS-GFP (AmpR)	This study
MB2940	C3029 + pZE1psoxS-GFP (AmpR)	This study
MB2942	C2566 + pZE1pdps-GFP (AmpR)	This study
MB2944	C3029 + pZE1pdps-GFP (AmpR)	This study
MB4856	C2566 + pQE60-Grx1-roGFP2-His (AmpR)	This study
MB4857	C3029 + pQE60-Grx1-roGFP2-His (AmpR)	This study
MB4854	C2566 + pQE60-roGFP2-Orp1-His (AmpR)	This study
MB4855	C3029 + pQE60-roGFP2-Orp1-His (AmpR)	This study
MB5994	C2566 + pQE60 (AmpR)	This study
MB5995	C3029 + pQE60 (AmpR)	This study
**Plasmids**	**Features**	**Source**
pZE1psoxS-GFP	GFP under the regulation of soxS promoter, pBR322 origin, AmpR	[36]
pZE1pdps-GFP	GFP under the regulation of dps promoter, pBR322 origin, AmpR	[36]
pQE60-Grx-roGFP2-His	Grx1-roGFP2 fusion under the regulation of T5 promoter, pBR322 origin, AmpR	[21]
pQE60-roGFP2-Orp1-His	roGFP2-Orp1 fusion under the regulation of T5 promoter, pBR322 origin, AmpR	[21]

Culture growth conditions: Cells were grown in 5 ml LB in test-tubes at 30 °C in the presence of appropriate antibiotics and 100 μM IPTG. 1 ml of cells were harvested, normalized to OD 1 and cells were washed once in phosphate buffered saline (PBS). 200 μL of PBS-washed cells were transferred into a 96 well Corning clear-bottom plate and emission intensities at 510 nm were measured when excited at 405 nm and 488 nm, using the Spectromax M5 Microplate Reader. Cells harboring empty vector were used as blanks to account for background fluorescence.

GFP plate measurements: Overnight cultures normalized to OD 1 were serially diluted and 5 μL of cells were spotted. GFP fluorescence was visualized by Amersham Typhoon RGB Biomolecular Imager with 532 nm excitation laser and 526SP emission filter.

H_2_O_2_ and DTT microplate injection assays with biosensor expressing *E. coli* strains: *E. coli* wild type and SHuffle strains expressing roGFP2 fused probes (Grx1-roGFP2 and roGFP2-Orp1) were cultivated in LB medium with 100 μM IPTG overnight to induce expression of the probes. Cells were harvested, washed with Belitsky minimal medium (BMM), adjusted to an OD_500_ of 2 in BMM, and transferred to microplate wells. The OxD of the biosensor cells were determined after injection of different doses of H_2_O_2_ in the wild type strain and DTT in the oxidized SHuffle strain. Samples for fully reduced and oxidized controls were treated for 10 min with 10 mM DTT and 5 mM diamide, respectively. The biosensor fluorescence emission was measured at 510 nm after excitation at 405 and 488 nm using the CLARIOstar microplate reader (BMG Labtech). The OxD was calculated based on the fluorescence intensities for each sample and normalized to fully reduced and oxidized controls as described below and in previous work [26].

Redox potential determination: The previously described equation [[27], [28]] (REF) was used to determine oxidation degree of the probe (OxD_roGFP2_), where R = ratio of the untreated probe value, R_red_ = ratio of fully reduced probe value, R_ox_ = ratio of fully oxidized probe value, *I*488_ox_/*I*488_red_ = ratio of fully oxidized probe at *I*488 divided by fully reduced probe at *I*488, which was experimentally calculated as approximately 0.35. The redox potential (*E*_roGFP2_) was calculated using the Nernst Equation, were *E*^*o*^_roGFP2_ = the standard midpoint potential of roGFP2 = −280 mV, and F = Faraday's constant of electric charge per mole of electrons, where 2 refers to the 2 electrons transferred during the redox reaction.

$$OxD_{roGFP2}=\frac{R-R_{red}}{\frac{I488_{ox}}{I488_{red}}\left(R_{ox}-R\right)+\left(R-R_{red}\right)}$$

$$E_{roGFP2}=E_{roGFP2}^{o'}-\frac{RT}{2F}In\left(\frac{1-OxD_{roGFP2}}{OxD_{roGFP2}}\right)$$

RNA-sequencing: SHuffle B cells (C3029) along with its parental wild type *E. coli* B strain (C2566) were grown in biological duplicates at 30 °C and samples were collected when OD_600nm_ reached 1.0. Total RNA of four cultures were prepared by using FastRNA Pro Blue kit (Qbiogene, catalog # 6025–050). DnaseI (NEB, M0303) was used to remove genomic DNA contamination, followed by its removal by heat inactivation in the presence of 5 mM EDTA. Qiagen RNeasy Column (Qiagen, Catalog #7404) was used to remove residual EDTA. Final yields were quantified using Qubit Fluorometer (RNA BR reagent). Ribosomal RNAs were depleted using NEBNext rRNA Depletion kit (NEB, E6310). Probes that bind to eukaryotic ribosomal RNAs were replaced by probes that bind to *E. coli* ribosomal RNAs. The RNA library was made with NEBNext Ultra Directional Kit (NEB, E7420). Library quality was analyzed by Agilent 2100 Bioanalyzer with Agilent High Sensitivity DNA kit (Agilent Technologies, catalog# 5067–4626). The average size of each cDNA library was about 350 bp. Equal amounts of the four different barcoded libraries were mixed and sequenced pair end 76 bp by Illumina NextSeq 500. Illumina sequencing data was analyzed by a workflow created in Galaxy including following steps: 1) SeqPrep (version 0.1) to trim read primer and adapter sequence, 2) FastQC (version 0.10.1) to assess the quality of raw sequence data, 3) Bowtie 2 (version 0.6) to align trimmed reads to reference genome (*E. coli* ER2566) [29] 4) SAM/BAM alignment summary metrics (version 1.56.0) to report high-level measures of alignment, 5) Down-sample SAM (version 1.56.0) to retain a random subset of the reads. The probability that any given read will be kept was set as 1; 6) Count the number of aligned the reads (a part of the bedtool package, version 0.1.0), 7) EdgeR (version 0.0.2) to determine differential expression.

AMS alkylation: Cells were grown in rich media supplemented with antibiotics until reaching late log phase of growth (5 h). OD_600nm_ was measured and cultures were diluted to the lowest OD. Three samples of 1 ml culture were incubated on ice for at least 15 min with 15% trichloroacetic acid (TCA). Cells were then subjected to alkylation by 4-acetamido-4′-maleimidylstilbene-2,2′-disulfonic acid (AMS) as previously described [30]. SDS-PAGE and Western blot were carried out by following standard protocols. In Western blot, the primary anti-His antibodies for detecting roGFP and the secondary antibodies were purchased from Cell Signaling Technology (catalog #2366 and #5257). The membrane was scanned with Li-Cor Odyssey Infrared fluorescent Imager.

Flow cytometry analysis: SHuffle cells (C3029) and parental wild type cells (C2566) were transformed with Grx1-roGFP2 and roGFP2-Orp1 expression plasmids and grown in LB supplemented with ampicillin until late log phase (OD600 = ~0.8), in three replicates. The cells were harvested and washed with PBS before flow cytometry analysis. Fully oxidized and reduced cells were determined through addition of 8 mM diamide, 100 mM H_2_O_2_ and 40 mM dithiothreitol (DTT), respectively for 5 min. Oxidation for 30 min with 100 mM yielded similar results. Flow cytometry analysis was done using sorting-equipped FACS Aria III flow cytometer (BD Biosciences, San Jose, CA) [31]. Briefly, 250 μL cells were analyzed using two lasers: 405 nm and 488 nm, a ratio between them was calculated for each cell, while dead and negative cells were gated out. Each sample had 10,000 cells. Distribution of the 405/488 ratio was derived for each sample and plotted using Excel.

## 3 Results

### 3.1 SHuffle cells are under oxidative stress

**Fig. 2:**
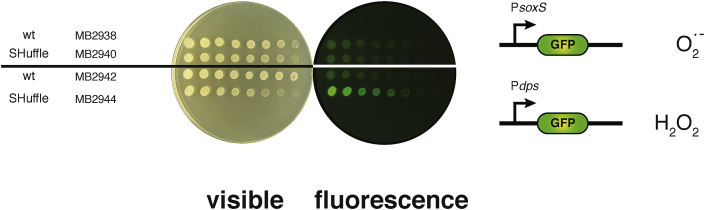
**SHuffle cells are under H**_**2**_**O**_**2**_**induced oxidative stress.** Cells expressing either the free radical reporter plasmid (wild type MB2938 and SHuffle MB2940) or the H_2_O_2_ reporter plasmid (wild type MB2942 and SHuffle MB2944) were serially diluted and spotted on rich plates. Plates were visualized either in visible light or using Typhoon scanner.

SHuffle cells have been engineered to promote oxidative folding of disulfide bonded proteins in its cytoplasm. This was achieved by genetically deleting the *gor* and *trxB* genes and selecting for a mutant *ahpC* that can reduce Grx1. The resulting mutant AhpC* has lost its capacity to reduce H_2_O_2_. The cumulative effect of these changes is postulated to result in cells that are subjected to oxidative stress due to loss of peroxidase activity of AhpC* and the lack of active thioredoxins (Trx1, Trx2) and glutaredoxins (Grx2, Grx3) [3]. In order to confirm that SHuffle cells are indeed under oxidative stress, two GFP-based reporter plasmids were used. The first reporter plasmid (pZE1psoxS-GFP) expresses GFP under the control of the *soxS* promoter, which is induced in the presence of redox active compounds, such as superoxide anions (O_2_^•−^) [32]. The second reporter plasmid (pZE1pdps-GFP) expresses GFP under the control of the *dps* promoter, which is induced in the presence of elevated levels of H_2_O_2_ [33]. SHuffle cells (C3029) and parental wild type cells (C2566) were transformed with pZE1psoxS-GFP or pZE1pdps-GFP. Transformants (MB2938, MB2940, MB2942 and MB2944) were grown in rich media with the appropriate antibiotics and 0.1 mM IPTG, their OD's were standardized and cells were spotted on rich agar plates and incubated overnight at 30 °C. Cell were visualized under visible light and by scanning with Typhoon RGB scanner to capture GFP fluorescence. Only SHuffle cells expressing GFP under the regulation of *dps* promoter displayed higher level of fluorescence in comparison to wild type cells. These results indicate that SHuffle cells are subjected to elevated levels of H_2_O_2_ but not superoxide anions (Fig. 2).

### 3.2 OxyR regulon is upregulated in SHuffle cells

To validate the previous observation that SHuffle cells are under H_2_O_2_-driven oxidative stress, transcriptional analysis of the OxyR and SoxRS regulons were conducted based on RNA-seq data on SHuffle (C3029) and parental wild type cells (C2566). For the purpose of this study, only the OxyR and SoxRS regulons were analyzed.

**Fig. 3:**
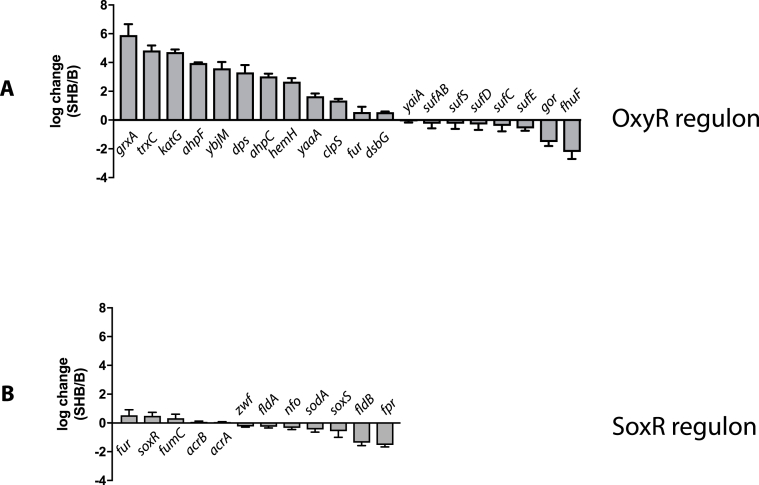
**Change in transcriptional levels of genes in OxyR (A) and SoxRS (B) regulons in SHuffle cells (C3029) compared to wild type cells (C2566).** Relative amounts of transcripts were quantified using RNAseq data and plotted as log difference between wt and SHuffle cells.

By comparing the number of reads in the previously characterized OxyR and SoxRS regulons [34] in SHuffle vs wild type cells, the genes whose transcripts were upregulated or downregulated were identified (Fig. 3). Of the 20 genes within the OxyR regulon [35], the majority of the genes were two to sixteen-fold upregulated in SHuffle cells, when compared to the wild type parental strain. The *dps* gene was 8-fold upregulated in SHuffle cells, supporting the previous observation of induced levels of *dps* promoter (Fig. 2). Transcripts for the *gor* gene were missing as *gor* is deleted in SHuffle and thus appear to be downregulated, while other studies have shown *fhuF* and *yaiF* to be not under OxyR regulon [34]. Intriguingly, although previous studies have confirmed that the *suf* operon responsible for iron-sulfur cluster biogenesis is under OxyR regulation [35], no upregulation of the *suf* operon was observed in SHuffle cells (Fig. 3A). One possible explanation was found by interrogating the genome of SHuffle cells. Analyzing the genome of SHuffle cells [29], revealed that the 3′ end of *sufA* and the 5′ end of downstream *sufB* genes have been deleted, resulting in a chimeric *sufAB* fusion gene. Furthermore, two nucleotides within the 5’ UTR region was altered when compared to parental wild type cells (data not shown). These observations suggest a significant genomic rearrangement of *sufA*, *sufB* genes along with changes in the promoter region, that may explain the lack of transcriptional response of the *suf* operon. In comparison to the *oxyR* regulon, the *soxRS* regulon genes were not upregulated (Fig. 3B), supporting the previous observation of the uninduced *soxS* promoter (Fig. 2). These results suggest that SHuffle cells suffer from endogenous H_2_O_2_ stress leading the constitutive expression of the OxyR regulon.

### 3.3 Expression and redox states of roGFP2 fused biosensors

To validate the expression levels of the roGFP2 fusion probes in *E. coli* wild type and SHuffle strains, Western blot analysis was conducted on soluble fractions of whole cell lysates using anti-His antibodies. In parallel, to investigate whether the redox-active cysteines of the biosensors are in their oxidized disulfide or reduced thiolate states, AMS alkylation of the cell extracts was performed. AMS alkylates free thiol groups, covalently adding 500 Da per cysteine, resulting in a mobility shift in SDS-PAGE analysis [30]. Western blot analysis revealed that both the roGFP2-Orp1 and the Grx1-roGFP2 fusions are expressed soluble to high levels, both in SHuffle and wild type cells.

**Fig. 4:**
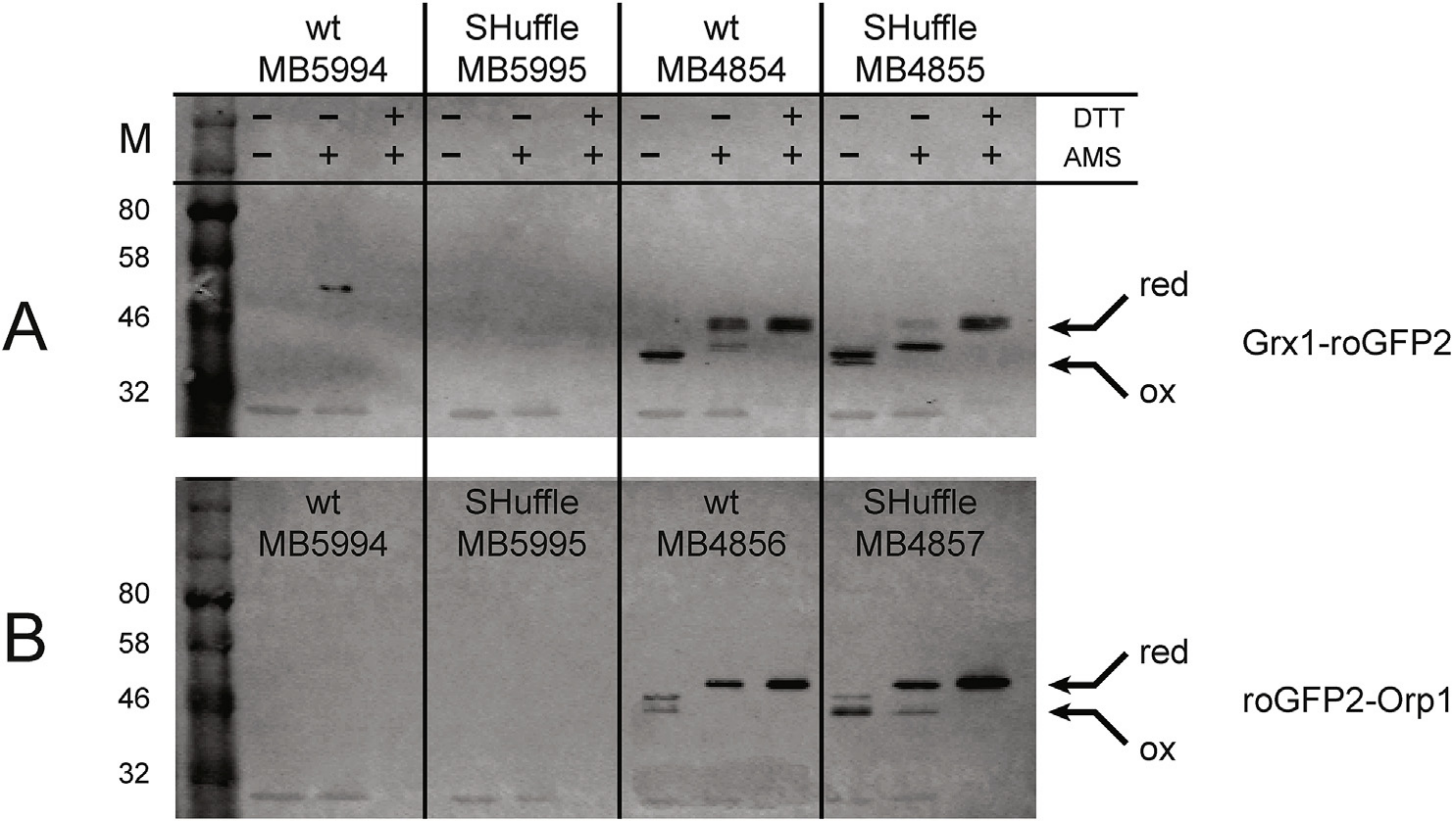
**Protein expression levels and redox states of roGFP2 fusion reporters in SHuffle and wild type cells.** Cells expressing either roGFP2-Orp1 (A) or Grx1-roGFP2 (B) were analyzed in a Western blot using anti-His antibodies. Redox states of the fusions were chemically interrogated using AMS. Samples treated with DTT served to identify the migration of the reduced (red) protein band while those not treated with AMS served to identify the oxidized band (ox), which are indicated with arrows. Wild type (MB5994) and SHuffle cells (MB5995) harboring empty vectors are shown.

The redox state of Grx1-roGFP2 fusion reporter was observed to be mostly reduced (~90% based on ImageJ analysis) with a minority (~10%) present as an AMS-resistant hemi-oxidized species when expressed in wild type cells. The reverse is observed when the Grx1-roGFP2 fusion reporter is expressed in SHuffle cells, with the hemi-oxidized species becoming the majority species (~90%) and the remainder being reduced. It is unclear whether the disulfide bonds in Grx1 or roGFP2 are oxidized when expressed in SHuffle. The redox state of the roGFP2-Orp1 fusion reporter was observed to be completely in its reduced state in wild type cells, whereas a small ~20% completely oxidized species can be observed when expressed in SHuffle cells. (Fig. 4).

### 3.4 Flow cytometry analysis roGFP2 probes in the SHuffle cell population

**Fig. 5:**
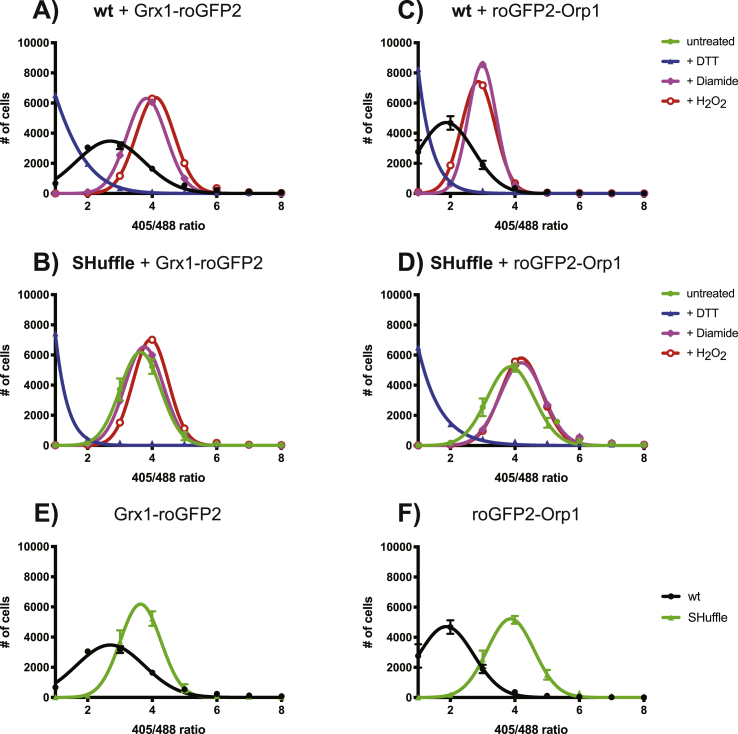
**Flow cytometry analysis of SHuffle and wild type cells expressing roGFP2 fusion reporters.** The fluorescence at 510 nm of wild type cells (black lines) expressing either Grx1-roGFP2 (A) or roGFP2-Orp1 (C), and SHuffle cells (green lines) expressing either Grx1-roGFP2 (B) or roGFP2-Orp1 (D) were measured using excitation at 405 nm and 488 nm lasers. Cells were counted (Y-axis) for having various ratios of 405nm/488 nm excitations and binned into 6 groups (X-axis). Fully oxidized samples were generated by treating the cells either with diamide (purple lines) or H_2_O_2_ (red lines), while fully reduced samples were treated with DTT (blue lines). Comparison of SHuffle vs wild type cells expressing either Grx1-roGFP2 (E) or roGFP2-Orp1 (F). Three biological replicated were used for treated and untreated samples. (For interpretation of the references to colour in this figure legend, the reader is referred to the Web version of this article.)

SHuffle and wild type cells expressing either roGFP2-Orp1 or Grx1-roGFP2 fusions were grown in rich media at 37 °C to OD_600nm_–0.8, harvested and washed with PBS. 10,000 cells were subjected to analysis with flow cytometry. For each cell, the ratio of fluorescence emission at 510 nm was measured when excited at 405 nm and 488 nm. In order to derive the OxD of the fully reduced and oxidized roGFP2 probes, cells expressing roGFP2 were treated with either 40 mM DTT, 8 mM diamide or 100 mM H_2_O_2_ for 5 min. An additional test of the H_2_O_2_ treated samples after 30 min resulted in identical results as that of 5 min, indicating that the cells were maximally oxidized. Short treatment was used in order to avoid cell death. To evaluate the OxD in the population of the parental and SHuffle strains, distribution of the 405/488 nm ratios of Grx1-roGFP2 was plotted relative to the distribution of either fully oxidized or reduced cells (Fig. 5).

Grx1-roGFP2 probes: Wild type cell expressing Grx1-roGFP2 displayed average excitation ratio of 3, which was in between the average excitation ratio of 2 and 4 for cells treated with DTT or diamide/H_2_O_2_ respectively (Fig. 5A). In contrast, SHuffle cells expressing the same Grx1-roGFP2 probe displayed average excitation ratios of 4, similar to cells treated with diamide or H_2_O_2_ (Fig. 5B).

roGFP2-Orp1 probes: Wild type cell expressing roGFP2-Orp1 displayed average excitation ratio of 2, which was in between the average excitation ratio of 1 and 3 for cells treated with DTT or diamide/H_2_O_2_ respectively (Fig. 5C). SHuffle cells expressing roGFP2-Orp1 displayed average excitation ratios of 4, similar to cells treated with diamide or H_2_O_2_ (Fig. 5D).

Both probes indicate that SHuffle cells are close to maximal oxidation states that roGFP2 fusions can report and are approximately on average 2-3-fold more oxidized than wild type cells (Fig. 5E and F). Meanwhile, the wild type cells are in between the fully reduced and oxidized states of both roGFP2-Orp1 and Grx1-roGFP2 probes (Fig. 5A and C). This data suggests that the roGFP2 probes are in the mid-point of their fully oxidized/reduced states *in vivo* and are ideally situated to sense perturbations to the redoxome of *E. coli*. Intriguingly, the average emission ratios of roGFP2-Orp1 probes expressed SHuffle cells (Fig. 5D) was 4, compared to 3 in wild type cells treated with diamide or H_2_O_2_ (Fig. 5C). This was not the case for Grx1-roGFP2 probes, where the average emission ratios in SHuffle cells and in wild type cells treated with diamide or H_2_O_2_ was 4 (Fig. 5A and B). Taken together, these results suggest that SHuffle cells are under greater H_2_O_2_-driven oxidative stress than wild type cells treated with diamide or H_2_O_2_, while the GSSG-driven oxidative stress in SHuffle cells is similar to what cells experience when treated with diamide or H_2_O_2_.

### 3.5 Oxidative stress of cells during growth

Using the roGFP2 probes, SHuffle cells have been demonstrated to be under oxidative stress, both from GSSG and H_2_O_2_. In order to understand the process of generating GSSG or H_2_O_2_ and their oxidative effects on roGFP2 probes, fluorescent emissions from roGFP2 probes expressed during growth phase was measured. SHuffle and wild type cells expressing either roGFP2-Orp1 or Grx1-roGFP2 fusions were grown in rich media at 30 °C for 24 h. Samples were then inoculated from overnight culture and grown in 5 ml of rich media with ampicillin selection. Cells were subsequently sampled every 3 h and re-suspended in 1 mL PBS at a normalized cell density (OD_600nm_ = 1). Ratios of oxidized vs reduced states of probes were measured at each time point by using the ratiometric emission of roGFP2 probes when excited at 405 nm and 488 nm, as described in methods.

**Fig. 6:**
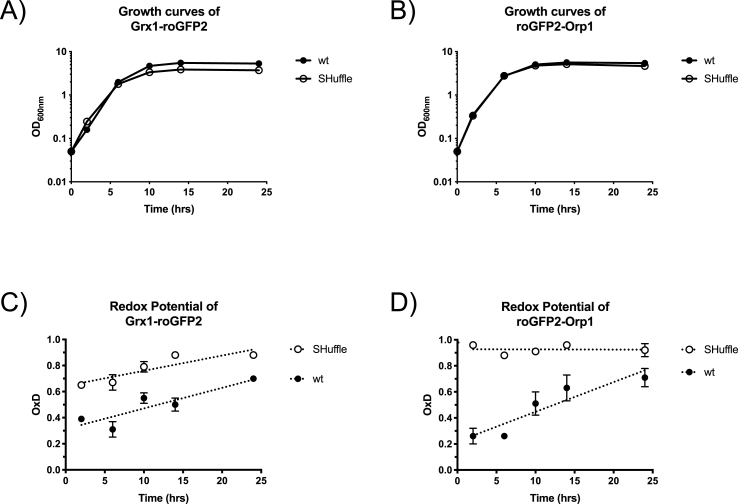
**Change in oxidation state of the roGFP2 probes in cells during growth.** Wild type (filled circles) or SHuffle cells (open circles) expressing either Grx1-roGFP2 (A and C) or roGFP2-Orp1 (B and D) were monitored for their growth at OD600nm (A and B) and the ratiometric response of the probes (OxD) was calculated based on the 400/488 nm excitation ratio, with emission measured at 510 nm (C and D).

Both SHuffle and its parental wild type cells grew very similarly at 30 °C (Fig. 6A and B). In agreement with previous data, both roGFP2-Orp1 and Grx1-roGFP2 probes showed higher states of oxidation at all stages of growth phase in SHuffle cells, when compared to wild type cells. Most informatively, both wild type and SHuffle cells accumulated oxidized Grx1-roGFP2 over time, reaching the maximum oxidized state in stationary phase (Fig. 6C and D). In contrast, SHuffle cells maintain maximum oxidized state when expressing roGFP2-Orp1 throughout the growth phase, while wild type cells reached their maximal oxidized states in stationary phase (Fig. 6C). These results indicate that both wild type and SHuffle cells accumulate oxidized glutathione slowly as the cultures age and enter stationary phase. However, wild type cells are initially rich in reduced glutathione and oxidation to its GSSG state takes time, whereas SHuffle cells are under constant H_2_O_2_-driven oxidative stress, so minimal change in redox state is observed over time.

Response of roGFP fusion probes to reduction/oxidation within living cells.

**Fig. 7:**
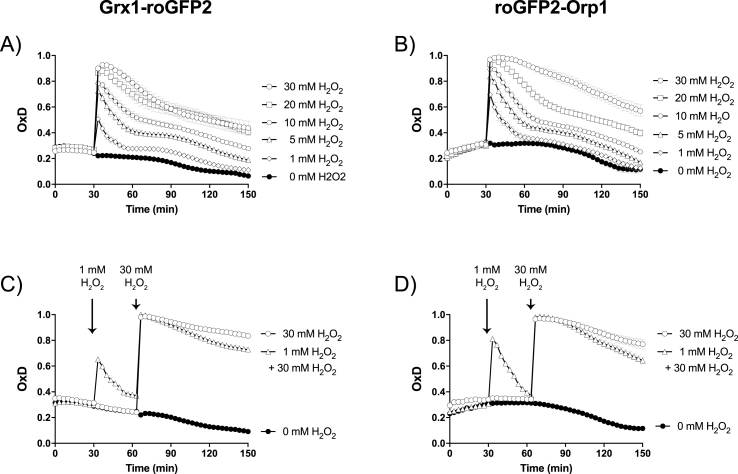
**Response of redox probes to H**_**2**_**O**_**2**_**in wild type cells.** Response to increasing concentrations of H_2_O_2_ of Grx1-roGFP2 (A and C) and roGFP2-Orp1(B and D) in wt cells (C2566) where measured using the CLARIOstar microplate reader (BMG Labtech). Injection of H_2_O_2_ was performed into microplate wells containing *E. coli* biosensor cells of an OD_500_ of 2. Samples for fully reduced and oxidized controls were treated for 10 min with 10 mM DTT and 5 mM diamide, respectively. The OxD was calculated using the 405/488 excitation ratio with emission at 510 nm.

In this study, roGFP2 fusion probes were used to study the changes of redox state in cells over time and to measure the redox-state of SHuffle cells vs wild type (wt) cells. To further gauge the *in vivo* response of the roGFP2 to oxidation, wt cells expressing either roGFP2-Orp1 or Grx1-roGFP2 were subjected to various concentrations of H_2_O_2_, and the ratiometric emission of the probes were measured over time. These experiments could only be conducted in wt cells, as the probes are at their fully oxidized in SHuffle cells (Fig. 5). Cells exposed H_2_O_2_ displayed a rapid response in the ratiometric emission of both roGFP2-Orp1 and Grx1-roGFP2. Based on the fluorescent response of the probes, the oxidizing H_2_O_2_ was rapidly reduced within the first 30 min by the reducing system of wt cells, followed by a slower reducing rate. Approximately 2 h after exposure, cells returned to their reduced state when exposed up to 10 mM H_2_O_2_ and could tolerate up to 30 mM H_2_O_2_, with slower recovery (Fig. 7A and B). We further analyzed if roGFP2 fusion probes can measure priming responses of *E. coli* to sub-lethal H_2_O_2_ challenge to induce improved tolerance to survive subsequent lethal H_2_O_2_ doses compared to non-primed cells. *E. coli* wild type cells primed with 1 mM H_2_O_2_ displayed a significant faster rate of recovery of the reduced state of the probes after subsequent exposure to 30 mM H_2_O_2_ (Fig. 7C and D) compared to non-primed *E. coli* cells, only exposed to the lethal dose.

**Fig. 8:**
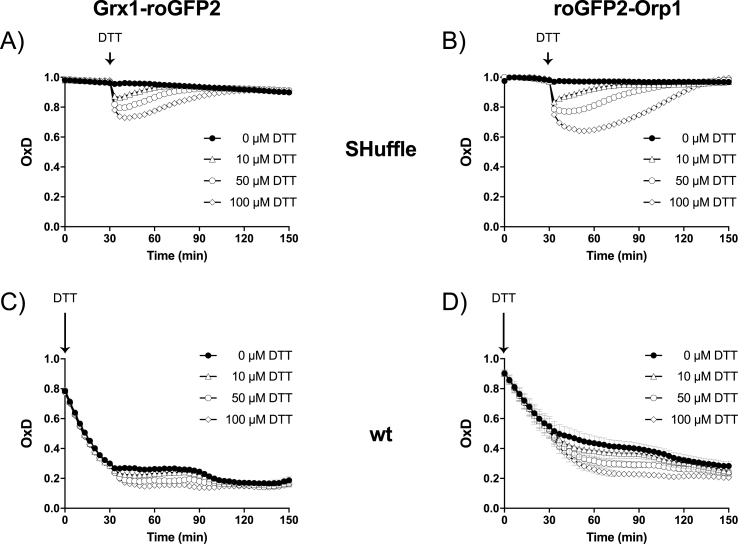
**Response of redox probes to DTT in wild type and SHuffle cells.** Response to increasing concentrations of DTT of Grx1-roGFP2 and roGFP2-Orp1expressed in SHuffle cells (A and B) or wild type cells (C and D) were measured using the CLARIOstar microplate reader (BMG Labtech). DTT injection was performed into microplate wells containing *E. coli* SHuffle biosensor cells or diamide-oxidized wild type cells of an OD_500_ of 2. Samples for fully reduced and oxidized controls were treated for 10 min with 10 mM DTT and 5 mM diamide, respectively. The OxD was calculated using the 405/488 excitation ratio with emission at 510 nm.

In similar fashion, SHuffle cells expressing either roGFP2-Orp1 or Grx1-roGFP2 were exposed to various concentrations of DTT and the re-oxidation of the probes in the cytoplasm of SHuffle cells were followed over time. SHuffle cells could tolerate up to 100 μM DTT and re-oxidize the Grx1-roGFP2 back to its fully oxidized state within 30 min to 1.5 h, when exposed to 10 μM or 100 μM DTT, respectively (Fig. 8A). However, the re-oxidation of the roGFP2-Orp1 probe when exposed to 10 μM or 100 μM DTT was significantly slower, resulting in approximately a 30 min time delay in recovery when compared to the Grx1-roGFP2 probe (Fig. 8B). The difference in re-oxidation between the two probes supports the previous observation that SHuffle cells are under greater H_2_O_2_-driven oxidative stress when compared to GSSG-driven oxidative stress (Fig. 6C and D). However, these differences in recovery were not observed in wild type cells. Wild type *E. coli* cells were initially exposed to 2.5 mM diamde for 5 min to generate fully oxidized probes. Diamide was removed by washing of cells with Belitsky minimal medium (BMM). Wild type *E. coli* cells expressing either roGFP2-Orp1 or Grx1-roGFP2 were subsequently exposed to various concentrations of DTT and the OxD of the probes was measured over time (Fig. 8C and D). Both probes displayed similar rates of slow reduction by DTT, reaching maximal reduced states within approximately 30 min.

Taken together, the roGFP2 fusion probes operate within biological parameters. They can be used not only to detect the *in vivo* redox state of the cytoplasm of both SHuffle and wt cells, but also can be used to measure the recovery of cells, when exposed to reducing/oxidizing agents.

## 4 Discussion

Measuring redox-coupled reactions with minimal disruption is necessary for a holistic understanding of biological reactions within a living cell. In order to achieve this, redox-sensitive GFP probes were used to gauge redox states *in vivo* in wild type and genetically engineered SHuffle cells. SHuffle has disrupted reducing pathways to generate active disulfide bonded proteins, resulting in oxidative stress. Transcriptional analysis and promoter reporters confirmed that the majority of the oxidative stress the cells experience is due to elevated levels of H_2_O_2_, resulting in the upregulation of the OxyR regulon. These observations were further confirmed with the use of the H_2_O_2_ specific roGFP2-Orp1fusion protein reporter, indicating that SHuffle cells are under constant H_2_O_2_-driven oxidative stress. While both SHuffle and wt cells experience increasingly stronger GSSG-driven oxidative stress over the growth period of the cells, probes reached their maximal oxidized state in deep stationary phase, as reflected through Grx1-roGFP2 redox measurements of the GSSG/GSH ratio over time. Supporting our observation, genes involved in protection against oxidative stress are highly upregulated in stationary phase [37], such that DNA-binding protein (Dps) protecting DNA from oxidative damage, becomes the most abundant protein in stationary phase [38].

The difference in pools of H_2_O_2_ vs GSSG may be due to the fact that the protein AhpF, responsible for maintaining the peroxidase in its active reduced state, is functional in SHuffle, while glutathione reductase (*gor*) has been deleted, resulting in cells that are incapable of reducing glutathione pools. Whether AhpF interacts and reduces the yeast peroxidase Orp1 remains to be shown. Since it has been demonstrated that Orp1 is reduced by thioredoxin in its native yeast host [39], it may also be reduced by thiol-reductases in *E. coli*, as Orp1 has been used in many diverse organisms [40]. The fact that the majority of the roGFP2-Orp1 fusion remains reduced when expressed in SHuffle indicates that the functional reductase such as AhpF, may in fact reduce the yeast peroxidase Orp1. Similarly, whether AhpC*, which has been shown to reduce glutathionylated Grx1 [5], interacts with the human Grx1 in the Grx1-roGFP2 probe is equally unknown. Since no fully oxidized species of Grx1-roGFP2 was observed when expressed in SHuffle cells, this may indicate that some basal level of reduction is occurring, perhaps by AhpC*. Although the roGFP2 fusion probes used in this study have been useful in demonstrating several redox properties of SHuffle cells, they have also highlighted several remaining questions. The *in vivo* redox state of the roGFP2 fusion probes at steady state levels measured through AMS alkylation indicate that the Grx1-roGFP2 probe is approximately 0% fully oxidized, 90% hemi-oxidized and 10% fully reduced while the roGFP2-Orp1 probe is approximately 10% fully oxidized and 90% full reduced (Fig. 4). Currently it is not possible to known which cysteines in the fusion partner or the roGFP2 are oxidized. These difference in their redox state indicate at minimum that the two roGFP2 fusion probes have differential interactions with the redox components of SHuffle cells. In addition, the relationship between the steady-state level of disulfide oxidation and fluorescence may not be straightforward - for example, the reduced state of the roGFP could be in a protein complex *in vivo* that quenches fluorescence.

Upon confirmation of the *in vivo* expression, redox state and functionality of roGFP2-Orp1 and Grx1-roGFP2 fusions, the fluorescent probes reported that SHuffle cells are approaching the maximal oxidation state of the roGFP2 fusions. This suggests that future engineering of SHuffle cells for improved oxidative capacity may not be achieved by furthering the oxidative state of the cytoplasm, but instead by engineering novel oxido-reductases with improved catalytic features and enhanced substrate specificity. It is also possible that the maximal oxidation state roGFP2 fusion probes can report is not broad enough and other more sensitive probes are required to be engineered. One potential application of the roGFP2 fusion probes are to sort for cells with an increased cytoplasmic redox potential. However, since the probes are fully oxidized when expressed in SHuffle cells (Supplementary Fig. 1), it is not possible to select for SHuffle cells with an increased cytoplasmic redox potential. However, the response of the roGFP2 fusion probes to exogenously added chemical oxidants/reductants, indicate their potential use to report mutant version of *E. coli* with lower capacity to reduce (for SHuffle cells) or oxidize (for wt cells) proteins.

The use of roGFP2 fusion probes can also permit the determination of the redox potential of the cytoplasm of both the genetically redox-engineered SHuffle cells and their parental isogenic wild type strains. Redox potentials are commonly used in the thiol:disulfide exchange literature but their extrapolation to biology needs precaution. The electrochemical potential of a compound simply describes its tendency to become oxidized, compared to a reference known as “standard hydrogen electrode”, where the reversible oxidation of hydrogen molecule to protons and electrons by a platinum electrode is given a redox potential of zero [41]. Most of the Trx-fold proteins participate in thiol:disulfide exchange reactions, either reducing or catalyzing the formation of disulfide bonds. Thioredoxin 1 of *E. coli* (Trx1, encoded by the gene *trx1*) is the best-studied representative of this family and it is usually considered a reducing protein because it has a redox potential of −270 mV [42], i.e. it has a high tendency to became oxidized in thiol:disulfide exchanges. Although the interpretation of redox potential measurement within living cells *in vivo* should be approached with caution, they can guide the prediction of the flow of electrons; e.g. electrons are predicted to flow from NAPDH (−370 mV) to Trx1 (−270 mv), as it is favorable because of the higher (more positive) redox potential of Trx; resulting in a higher tendency to gain electrons.

**Fig. 9:**
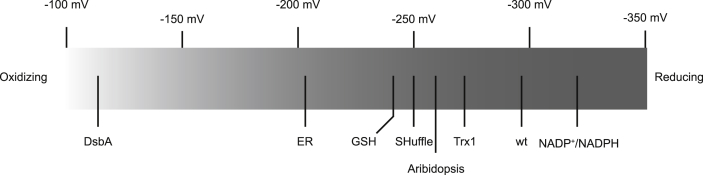
**Redox state of the cytoplasm of wild type and SHuffle cells.** Redox states measured by roGFP probes of *E. coli* SHuffle (−250 mV) and wild type (−291 mV) cells are shown in comparison to previously measured redox states of DsbA (−110 mv) [43], endoplasmic reticulum (−180 mv) [44], GSH (−240 mV) [45], *Arabidopsis* seedlings (−260 mV) [46], Trx1 (−270 mv) [42] and NADP+/NADH (−320 mV) [47].

Using the ratiometric fluorescence of the roGFP2-fusion probes, the redox potential of SHuffle cells using Grx1-roGFP2 was approximately −260 mV, while the maximum redox potential reached by the roGFP2-Orp1 probe was −250 mV. However, in wild type cells, both roGFP2 probes reached the maximum redox potential of approximately −280 mV. Comparing the redox states measured in this study with other redox states of cellular compartments and enzymes, indicated that although SHuffle cells are significantly more oxidizing than the cytoplasm of wild type cells, it was still significantly more reducing than previously measured redox states of the oxidizing eukaryotic endoplasmic reticulum (Fig. 9).

Although the use of roGFP2 probes expands our understanding of the complex redox process within living cells, many questions remain unanswered. Even though detailed *in vitro* characterization of the probes has been conducted [23], the process that oxidizes the roGFP2 probes within the cytoplasm of SHuffle cells remains to be demonstrated. For example, roGFP2-Orp1 probe is more oxidized in SHuffle cells than in wild type cells treated with the oxidants diamide or H_2_O_2_. One possible explanation is that wild type cells reduce and protect cells against diamide/H_2_O_2_ immediately and efficiently lower maximal oxidation, while GSSG-induced oxidation cannot be reduced as efficiently.

In conclusion, this study has demonstrated that the complex redoxome of SHuffle cells with disrupted redox pathways, can be monitored *in vivo* with minimal disruption to the cells. The roGFP2 based probes greatly enhance the capacity to monitor redox reaction within living prokaryotic cells and opens the path to develop further genetic mutagenesis schemes to engineer and select strains with novel redox properties.
