# Supplementary figures and images for: Utilizing redox-sensitive GFP fusions to detect in vivo redox changes in a genetically engineered prokaryote
Source: Redox Biol. 2019 Jul 20;26:101280. doi: 10.1016/j.redox.2019.101280 (PMC6831853; doi:10.1016/j.redox.2019.101280)

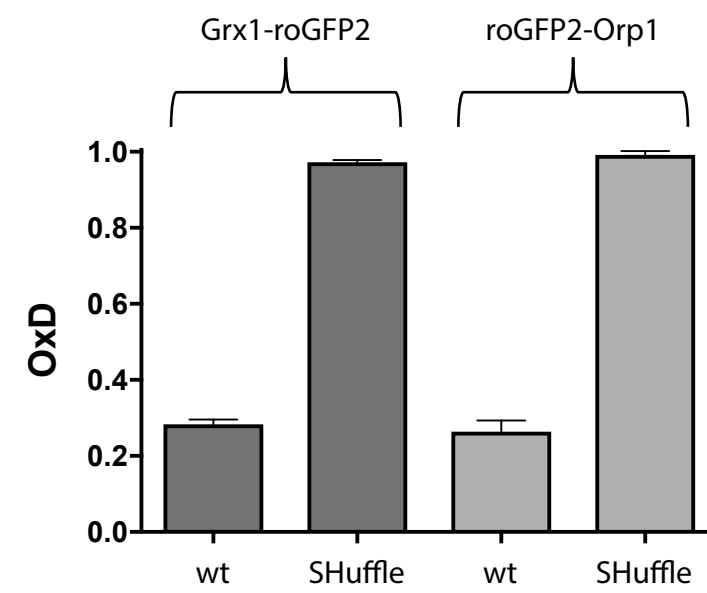

**Supplementary Figure 1.**roGFP2 probes are fully oxidized when expressed in SHuffle cells.

Supplement: Multimedia component 1 [file mmc1.pdf]
